# Phylogenomic Analysis of Deep-Branching Telonemid

**DOI:** 10.1093/gbe/evaf202

**Published:** 2025-10-29

**Authors:** Saelin Bjornson, Gordon Lax, Noriko Okamoto, Patrick J Keeling

**Affiliations:** Department of Botany, University of British Columbia, Vancouver, Canada; Department of Botany, University of British Columbia, Vancouver, Canada; Department of Botany, University of British Columbia, Vancouver, Canada; Department of Botany, University of British Columbia, Vancouver, Canada

**Keywords:** Telonemia, Hemimastigophora, TSAR, eukaryotic evolution, phylotranscriptomics

## Abstract

The evolutionary history of eukaryotic supergroups has been investigated primarily by large-scale phylogenomics, but one major hindrance to continued progress is that some major eukaryotic groups have extremely sparse sampling. The phylum Telonemia is one such group. Environmental sampling shows two major subgroups of Telonemia, but there are only multigene phylogenomic data from three closely related species belonging to one of the subgroups, TEL1. Here, a single cell was isolated from the pelagic Pacific Ocean, which SSU phylogenetic analysis reveals to be a telonemid of the TEL2 subgroup and distantly related to telonemids with multigene sequence data. Through single-cell transcriptome sequencing and phylogenomic analysis, we investigate the impact of this new telonemid on the relation of Telonemia to the Stramenopila-Alveolata-Rhizaria supergroup (SAR) and other sparsely sampled or historically unstable supergroups, namely, Hemimastigophora, Provora, and Haptista. Our maximum-likelihood (ML) analysis supports Telonemia as sister to Hemimastigophora, together as sister to SAR, with Haptista and Provora forming a clade and sister to all three. However, our Bayesian analysis failed to converge on a topology. Throughout different Telonemia sampling, gene sampling, alignment trimming, and site removal schemes, the sisterhood of Telonemia and Hemimastigophora remained largely supported in ML trees, even when their sisterhood to SAR dissolved, as was the sisterhood of Haptista and Provora. Inclusion of our TEL2 telonemid largely did not influence these relationships. However, our results also highlight the unstable placements of aforementioned groups throughout variations of our data, of which subsets give results consistent with other previously published analyses of this scale.

Significance A confident understanding of eukaryotic supergroup relationships is a prerequisite to study the deep evolutionary history of eukaryotes. A crucial shortcoming of multigene phylogenomic analyses of eukaryotic supergroups is the lack of taxon sampling in many major lineages. An example of this is the Telonemia phylum, of which only three closely related species have adequate sequence data for phylogenomic analysis. This study presents transcriptomic data from a fourth, early-diverging telonemid cell. We investigate the placement of Telonemia within eukaryotes, including the robustness of this placement throughout variations of data processing and subsampling. Our results support an alternative placement of Telonemia with group Hemimastigophora, together sister to SAR, conflicting with some previous studies.

## Introduction

The phylum Telonemia is a historically poorly represented group of microbial eukaryotes. For nearly a century, only a single species was known: *Telonema subtilis* (Griessmann 1913), with the second species, *T. antarcticum*, only described in 2005 (Klaveness et al. 2005), and later renamed *Lateronema antarcticum* (Cavalier-Smith et al. 2015). Environmental SSU rDNA sequences have nevertheless revealed a cosmopolitan distribution for both marine and freshwater telonemids (Shalchian-Tabrizi et al. 2007), and SSU phylogenies suggest ∼20 subgroups falling into two major subdivisions: TEL1 including *T. subtilis* and TEL2 including *L. antarcticum* (Shalchian-Tabrizi et al. 2007; Bråte et al. 2010; Tikhonenkov et al. 2022a). Six additional species were recently described (Tikhonenkov et al. 2022a), all belonging to the TEL1 clade, so *L. antarcticum* remains the only described species in the TEL2 clade.

Multigene phylogenomic analyses are currently restricted to three telonemid species in the TEL1 clade: *Telonema subtilis*, *Arpakorses versatilis*, and *Arpakorses idiomastiga*. This is unfortunate because all data show telonemids to be only distantly related to other eukaryotes, and their specific relationships to other major eukaryotic groups remain contentious. Recent multigene phylogenies including TEL1 species suggest they are sister to the SAR supergroup (Stramenopila, Alveolata, and Rhizaria), leading to the proposal of a TSAR supergroup (Strassert et al. 2019; Schön et al. 2021). However, other analyses place Telonemia as sister to Hemimastigophora (Tice et al. 2021) or with Haptista (Haptophyta + Centroheliozoa) (Eglit et al. 2024), either of which together branch as sister to SAR. Hemimastigophora is a similarly poorly represented group, with sequence data from only two species available (Lax et al. 2018) and multigene phylogenies placing them as sister to telonemids (Tice et al. 2021), but alternatively as sister to Cryptista, Archaeplastida, Haptista, and TSAR collectively or to Haptista and TSAR (Lax et al. 2018). Haptista has also been found to be related to TSAR (Irisarri et al. 2021; Tikhonenkov et al. 2022b; Eglit et al. 2024) or Cryptista and Archaeplastida (Schön et al. 2021; Tice et al. 2021). Another new group, the Provora, has also been found to be sister to Haptista and TSAR in a study that summed up this confusing situation by showing several conflicting topologies among Hemimastigophora, Haptista, Provora, Telonemia, and SAR among maximum-likelihood (ML) and Bayesian Inference (BI) trees (Tikhonenkov et al. 2022b).

Telonemids are one focal point of these debates, and the lack of data from diverse members of the group is a potential source of bias. Here, we reexamine the position of telonemids using the first available transcriptome from a representative of the TEL2 clade, derived from a single cell manually isolated from pelagic water from the Pacific Ocean. We show that TEL1 and TEL2 are monophyletic and together form a highly supported clade with Hemimastigophora, both branching sister to SAR. We also examine the impacts of gene sampling, trimming algorithms, Telonemia subsampling, and removal of fast-evolving sites.

## Results and Discussion

### Cell Telonemia sp. DSEL18 Is a Member of the TEL2 Clade

A single cell (DSEL18) with the overall appearance of a telonemid (Fig. S1) was isolated from water collected at 190 m depth in Monterey Bay and had its transcriptome sequenced. An SSU rRNA ML phylogeny including sequences from diverse Telonemia subgroups (Shalchian-Tabrizi et al. 2007; Bråte et al. 2010; Tikhonenkov et al. 2022a) confirmed this cell to be a telonemid (Fig. S2). DSEL18 fell in the 2j subgroup of TEL2 (65% bootstrap support (BS)), making it the only taxon in TEL2 with transcriptome data and distinct from the only species in TEL2 that has been described, *L. antarcticum*, a member of subgroup 2k (Fig. S2).

### Phylogenomic Placement of TEL2 Telonemids

**Fig. 1.**
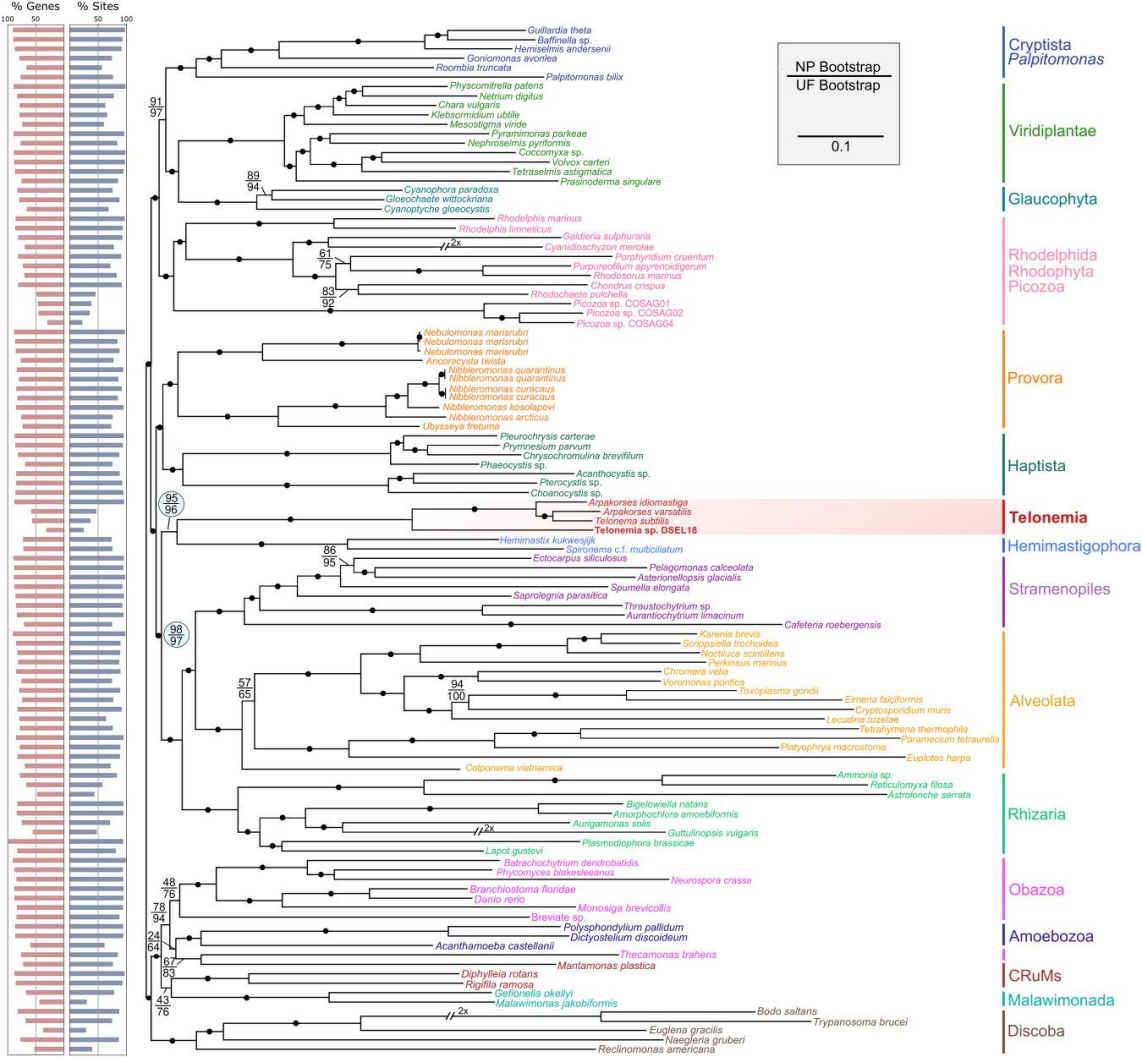
Maximum-likelihood tree inferred from 233 BMGE-trimmed proteins and 109 taxa under the LG + C60 + F + R + PMSF model of evolution, with LG + C20 + F + R used as the guide tree. Node values show nonparametric bootstrap support out of 200 (top) and ultrafast bootstrap support out of 1,000 (bottom). Circles represent ultrafast bootstrap support of ≥98% and nonparametric bootstrap support of ≥90%. Scale bar represents estimated amino acid substitutions per site. Branches labeled with 2× have been scaled to half their original length. Bar graph on the left shows the percent of genes and individual sites of each taxon.

For phylogenomic analyses, we first selected 109 eukaryotes and 233 proteins (see Materials and Methods), of which 78/233 (33%) were present in Telonemia sp. DSEL18, resulting in the final concatenated alignment with 13,687 out of 57,444 sites (24%) present in this taxon when alignments are trimmed with Block Mapping and Gathering with Entropy (BMGE), which removes high-entropy sites (Criscuolo and Gribaldo 2010) (Fig. 1), or 15,196 of 73,229 sites (21%) present when trimmed with trimAl, which removes sites by gap thresholds (Capella-Gutiérrez et al. 2009) (Fig. S3). The overall topology of ML trees based on this data is largely congruent with many previously published phylogenomic studies, with a few notable exceptions. One exception is the clade composed of apusomonad *Thecamonas trahen*s and CruMs member *Mantamonas plastica*. In previous analyses, Apusomonadida are within the supergroup Obazoa, and CruMs are monophyletic and sister to both Obazoa and Amoebozoa (Brown et al. 2018; Heiss et al. 2018; Tice et al. 2021; Tikhonenkov et al. 2022b). A note on this inconsistency is included in the Supplementary material. Another exception is that Provora is a sister to Haptista, which is inconsistent with previous analyses (Tikhonenkov et al. 2022b) where Provora branched with Hemimastigophora in many analyses, although not in all.

The monophyly of telonemids was completely supported in our phylogenomic analysis (Fig. 1, Fig. S3), and this group branched with Hemimastigophora with high support (95% BS, 96% UFB). This clade was in turn sister to SAR with stronger support (98% BS, 97% UFB, collectively referred to as HT + SAR), similar to a topology reported previously based on analyses including three TEL1 telonemids (Tice et al. 2021).

### Testing the Placement of Telonemids

**Fig. 2.**
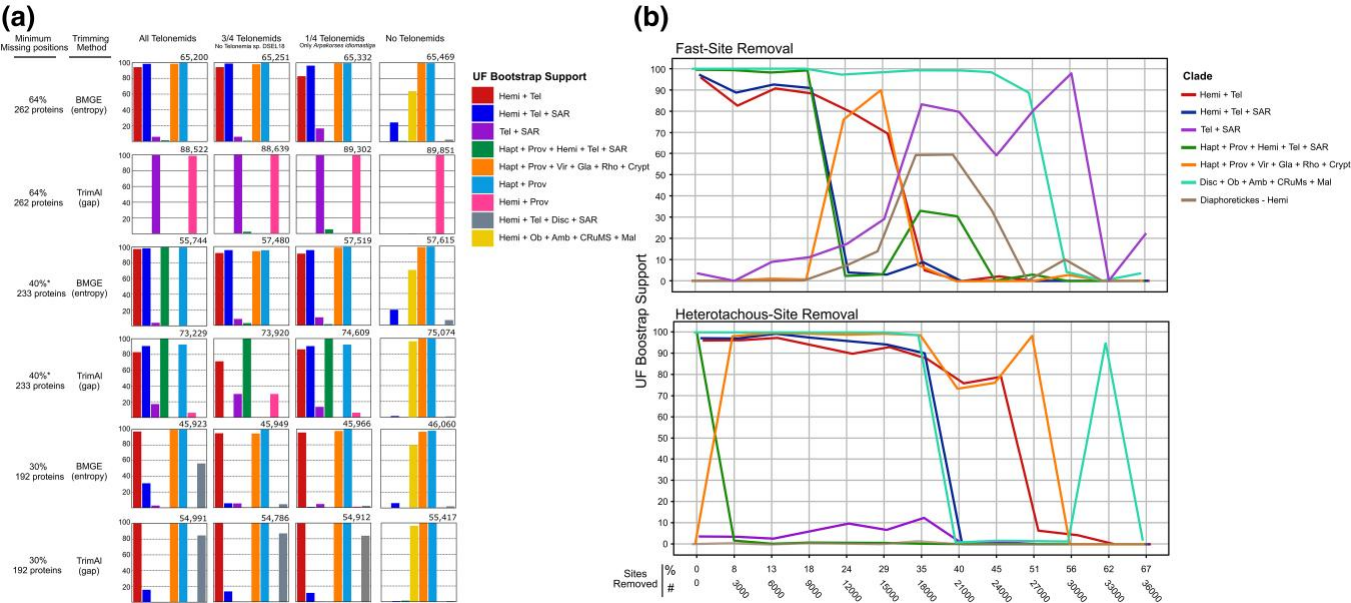
a) Impact of protein sampling, Telonemia sampling, and trimming algorithm on placements of various groups. Bars indicate summarized ultrafast bootstrap support (out of 1,000) of relevant nodes from ML trees inferred by LG + C60 + F + R + PMSF models, with LG + C20 + F + R used for the guide tree. Maximum missing positions refers to the threshold used for protein selection. Asterisks note that an additional four proteins were added past this threshold, with ≤50% missing positions in at least three out of four telonemids. Numbers above the graphs indicate the number of alignment sites. Tel, Telonemia; Hemi, Hemimastigophora; Hapt, Haptista; Prov, Provora; Crypt, Cryptista; Vir, Viridiplantae; Gla, Glaucophyta; Rho, Rhodophyta; Disc, Discoba; Ob, Obazoa; Mal, Malawimonada. When no telonemids are included, “Hemi + Tel” refers solely to Hemimastigophora. b) Stepwise removal of fast- and heterotachously evolving sites from an original alignment of 233 BMGE-trimmed proteins with 55,744 sites. Each step represents the removal of the top 3,000 sites. All trees were inferred under LG + C60 + F + R + PMSF models with 1,000 ultrafast bootstraps, with LG + C20 + F + R used for the guide tree.

Given the various positions found for telonemids in different published analyses, we re-examined the results by removing Telonemia sp. DSEL18, retaining only the telonemid with the highest coverage (96% site coverage), and removing all telonemids. In parallel, we also compared the effect of trimming alignments with BMGE or trimAl and of different site coverage thresholds for protein selection. ML analyses of most of these variants recovered H + T with high support, and inclusion of Telonemia sp. DSEL18 largely did not influence the placement of telonemids in our dataset (Fig. 2a). When H + T was favored, it was most often sister to SAR. Haptista was also most often supported as the sister to Provora. Notably, the choice of trimming method produced dramatic differences in placements of Hemimastigophora, Provora, and Haptophyta only when sparser genes were included—TrimAl-trimmed 262 gene datasets were the only ones to recover TSAR, Haptista + TSAR, and place Hemimastigophora sister to Provora (Fig. 2a, Figs. S4 to S7, Table S3).

Comparing these results to previous analyses that include at least Telonemia and Hemimastigophora (Lax et al. 2018; Tice et al. 2021; Tikhonenkov et al. 2022b; Eglit et al. 2024), we see a wide range of outcomes, with overlapping results in subsets of our variants and published analyses. For example, Haptista + TSAR and Hemimastigophora + Provora were both recovered in our largest datasets (>88,000 sites) and in Tikhonenkov et al. (2022b). Similarly, Lax et al. (2018) found Hemimastigophora sister to Haptista + TSAR or Hemimastigophora + Diaphoretickes depending on the data used, which both also appeared in our variant data sets. The results of Tice et al. (2021) match ours most consistently, as the HT + SAR relationship is recovered in most of our comparisons. Interestingly, despite this consistency, our dataset and that of Tice et al. (2021) contain less overlap (166 genes, 156 in telonemids) than those of Lax et al. (2018) and Tikhonenkov et al. (202 and 257 overlapped genes, 186 and 238 in telonemids, respectively) (Table S3). On the other hand, the results of Eglit et al. (2024), whose dataset is a subset of Lax et al. (2018), are most dissimilar, potentially due to their inclusion of the newly sequenced *Meteora sporadica*, which they placed sister to Hemimastigophora, and their lack of multiple Provora. In their study, Telonemia and Hemimastigophora placements differ between trees with different taxa, but in no case support TSAR or Hemimastigophora + Telonemia, nor was Haptista placed with provorid *Ancoracysta*.

We also examined UF bootstrap support over the stepwise removal of fast-evolving and heterotachously evolving (evolving under different rates across lineages) sites from our original 233-gene, BMGE-trimmed ML matrix (Fig. 2b). In both analyses, the H + T relationship was more robust to site removal than that of HT + SAR. In fast-site removal, TSAR becomes strongly supported after H + T falls in support at ∼30% site removal, whereas in heterotachous-site removal, TSAR is at no point highly supported. In heterotachous-site removal, the larger grouping of Haptista + Provora with HT + SAR loses support very quickly, after which Haptista and Provora are strongly supported as placed in a clade with Archaeplastida + Cryptista (Fig. 2b).

### Bayesian Inference

Using the 233-gene alignment subsampled to 81 taxa, Bayesian inference (BI) was conducted under the model CAT-GTR+Γ4. We ran four independent Markov chain Monte Carlo (MCMC) chains, and consensus trees were summarized from >15,000 trees (10% burn-in) (Fig. S8). However, no two chains converged, and placements of Telonemia, Hemimastigophora, Haptista, and Provora were all inconsistent. We then employed the more computationally efficient CAT-poisson+Γ4 model, summarizing from >10,000 trees (10% burn-in) (Fig. S2). Under this model, only chains 1 and 4 achieved a maximum difference less than 1 (maxdiff 0.39, meandiff 0.007). TSAR was supported in all chains (PP 1), and Haptista + TSAR converged in three out of four chains. Hemimastigophora + Diaphoretickes also converged in three out of four chains (posterior probability of 1).

## Conclusion

Overall, our results question the validity of the TSAR supergroup to the exclusion of Hemimastigophora, and more broadly demonstrate the instability of Telonemia, Hemimastigophora, Provora, and Haptista based on current datasets. These groups often branch near each other on the tree of eukaryotes, usually with high support, but topologies remain conflicting between different analyses.

One factor that emerges from these analyses is the length of the concatenations. Lax et al. (2018) and Tikhonenkov et al. (2022b) both include >90,000 sites and recover TSAR in both ML and BI analyses, while Tice et al. (2021) and Eglit et al. (2024), each with 70 to 73,000 sites, do not. Our ML analyses only recover TSAR in our longest concatenations, >88,000 sites. Choice of trimming can also influence results, and in our dataset is most influential when all genes are included—however, Lax et al. and Tikhonenkov et al. both use BMGE, while we recover TSAR in trimAl-trimmed matrices only. Additionally, our gene dataset, even when sampled to fewer genes, contains more overlap with these datasets than that of Tice et al. (2021), ruling this out as a source of bias toward the HT + SAR topology in shorter alignments.

An investigation by Irisarri et al. (2021) into the monophyly of Archaeplastida across datasets found that longer datasets resulted in more topological consistency than various subsets of genes and taxa. This trend may extend to inconsistencies found here. For instance, it is possible that longer datasets discussed here contain more phylogenetically informative sites that better counteract fast-site noise affecting TSAR support in shorter datasets, as in our 233-gene ML tree (Fig. 2b), TSAR is favoured over HT + SAR after removal of ∼35% of the fastest-evolving sites, and HT + SAR also falls in support in Tice et al. (2021) after removal of the ∼25% fastest-evolving sites. However, as nodes with sparse taxon sampling are particularly susceptible to nonphylogenetic signal, to which longer datasets are not impervious (Philippe et al. 2011), it is likely that trends will not become clear until denser taxon sampling is feasible in phyla that currently have few representatives, in combination with careful construction of ultra-long phylogenomic datasets.

## Materials and Methods

### Single-Cell Isolation, Sequencing, and Transcriptome Assembly

Telonemia sp. DSEL18 was isolated during a research cruise by the Monterey Bay Aquarium Research Institute. Water was sampled at 34.286N, −127.355W on 2018 January 30 at a depth of 190 m using an SBE 9plus conductivity–temperature–depth rosette sampler (Sea-Bird Electronics). A single cell of Telonemia was identified by microscopy and placed in 2 µL of Smart-Seq2 lysis buffer at −80 °C. cDNA was generated with the Smart-Seq2 protocol (Picelli et al. 2014), from which libraries were prepared with Illumina Nextera XT and sequenced on the Illumina NextSeq 550 platform. Reads were trimmed with Trimmomatic v0.39 (ILLUMINACLIP:TruSeq3-PE-2.fa:2:30:10:2:True LEADING:3 TRAILING:3 SLIDINGWINDOW:4:30 MINLEN:36) (Bolger et al. 2014) and assembled with rnaSPAdes v3.15.1 (Bushmanova et al. 2019). Transcript-coding regions were predicted using TransDecoder v5.5.0 (https://www.github.com/TransDecoder/), incorporating search results to UniProt Swiss-Prot and Pfam databases for coding region selection.

### Phylogenetic Analyses

To obtain ribosomal SSU rDNA sequences, Telonemia SSU sequences were collected from NCBI Genbank and used as blastn queries to search against our transcriptome. For phylogenetic analysis, additional Telonemia SSU sequences were collected from SILVA (Quast et al. 2012). Sequences were clustered at 99% identity using CD-HIT (Fu et al. 2012), aligned with MAFFT-LINS-I v7 (Katoh and Standley 2013), and trimmed with trimAl v1.2 (-gt 0.5) (Capella-Gutiérrez et al. 2009). ModelFinder (Kalyaanamoorthy et al. 2017) was run using IQ-TREE v2.1.0 (Minh et al. 2020), which selected the TN + F + R3 model of evolution, which was run with 1,000 nonparametric bootstraps.

For multigene analysis, we built upon a preexisting dataset of 263 proteins with representatives across eukaryote diversity (Burki et al. 2016; Hehenberger et al. 2017; Irwin et al. 2019; Mathur et al. 2019; Strassert et al. 2019), to which new taxa (see Table S1) were added to this dataset by blastp search of proteomes. Hits with an *e*-value of <10^−20^ and 50% coverage were retained and searched with blastp to the UniProt Swiss-Prot database. Regions that did not align to Swiss-Prot curated proteins were trimmed using a custom script. Single-gene alignments of the 263 proteins and candidate additions from new taxa were made with MAFFT-LINS-I v7 and trimmed with trimAl (-gappyout). Single-gene trees were constructed in IQ-TREE v2.1.0 using the LG + G model with 1,000 ultrafast bootstrap replicates (Minh et al. 2013), and each tree was visually inspected to remove contaminant, nonhomologous, and paralogous sequences from added taxa with FigTree (http://tree.bio.ed.ac.uk/software/figtree/). Clean sequences were aligned and trimmed as above, prior to calculation of taxon and protein alignment coverage using SCaFoS v1.2.5 (Roure et al. 2007). A set of 109 taxa was selected to accommodate phylogenetic breadth across eukaryotes and higher site coverage per taxon in alignments, after which sequences were again realigned.

In total, 24 final matrices were subjected to ML analyses. Four different taxa sets were used by including either all telonemids, excluding the lowest-coverage telonemid, including only the single highest-coverage telonemid, or excluding all telonemids. For each, single-protein alignments were trimmed with BMGE (-BLOSUM30) or trimAl (-gappyout). From each trimming method, three sets of proteins were chosen. A supermatrix of all proteins and all species was made with SCaFoS for both trimming methods, and thresholds were chosen according to these output statistic (“out.stat”) files. A “good” set contained proteins that had ≤40% missing positions across all taxa in one of the trimming methods, with an additional four with ≤50% missing positions in at least three out of four telonemids (233 proteins). A smaller set included only proteins with ≤ 30% missing positions (192 proteins). A larger set included all proteins in our dataset, excluding one very low-coverage protein (262 proteins). Selected proteins were then realigned and retrimmed with only selected taxa.

All ML analyses were done in IQ-TREE v2.1.0 under the evolutionary model LG + C60 + F + R + PMSF (Posterior Mean Site Frequencies; Wang et al. 2018) (-m LG + C60 + F + R -ft ), using guide trees generated by command -m LG + C20 + F + R -bb 1000, done separately for each tree inferred. Nonparametric bootstrap support was found for both BMGE- and TrimAl-trimmed 233-gene matrices. The BMGE 233-gene matrix was the starting matrix for removal of fast-evolving and heterotachously evolving sites, which were removed using PhyloFisher tools fast_site_remover.py and heterotachy.py (Tice et al. 2021) with default settings. Ultrafast bootstrap support for nodes of interest was found using the CONSENSE program from PHYLIP v3.697 (Felsenstein 1993) on IQ-TREE.UFBoot output files, generated by option --boot-trees. The BMGE 233-gene matrix was also used for BI analysis. For computational feasibility, a smaller subset of 81 species was used by selecting the best-represented taxa from each phylum. BI was conducted using PhyloBayes-MPI (Lartillot et al. 2013) under CAT-GTR+Γ4 (mpirun -np 32 pb_mpi –d -cat -gtr -S -dgam 4 ) and CAT-Poisson+Γ4 models (mpirun -np 32 pb_mpi -d -cat -poisson -S -dgam 4 ). Four MCMC chains were run simultaneously until each generated 15,000 or 10,000 trees, respectively, taking 2 to 3 months. Consensus trees were found by bpcomp, applying a 10% burn-in and subsampling every 10 trees. Larger burn-ins of up to ∼50% were attempted but did not affect the convergence of chains.

## Supplementary Material

evaf202_Supplementary_Data

## Data Availability

The data underlying this article are available in Figshare at: https://doi.org/10.6084/m9.figshare.28467455. Raw transcriptome sequence reads of Telonemia sp. DSEL18 have been deposited in the NCBI Sequence Read Archive under BioProject accession number PRJNA1159223.
