# Supplementary material for: Phylogenomic Analysis of Deep-Branching Telonemid
Source: Genome Biol Evol. 2025 Oct 29;17(12):evaf202. doi: 10.1093/gbe/evaf202 (PMC12676123; doi:10.1093/gbe/evaf202)
Supplement: evaf202_Supplementary_Data [file evaf202_supplementary_data.docx]

**Supplementary note on *Mantamonas* and *Thecamonas***

More inspection of the placements of obazoan member *Thecamonas* (apusomonad) and CRuMs member *Mantamonas* was warranted. This relationship is highly unstable in our dataset. Interestingly, our ML tree with 200 non-parametric bootstraps inferred from the 233-gene BMGE-trimmed dataset did not recover CruMs, however in re-running this dataset with 1,000 UF bootstrap support the same dataset did recover CRuMs (Fig S4). All ML trees were inferred under the LG+C60+F+R+PMSF model using the LG+C20+F+R model for a guide tree, done on each tree separately.

Of our various datasets (with different gene sampling, telonemia sampling and trimming methods) analysed with 1,000 UF bootstraps, CRuMs was sporadically monophyletic (Fig S4-S7, summarized in Table S2). CRuMs was only recovered once in trimAl-trimmed datasets, when only one telonemid was included in the 192-gene set. In BMGE-trimmed datasets, CRuMs was monophyletic in all gene sampling schemes when all telonemids or all but Telonemia sp. DSEL18 were sampled. At lower telonemia sampling (only *Arpakorses idomastiga* or no telonemids), CRuMs was recovered in 262- and 192-gene datasets but not in 233-gene datasets. As all genes in each dataset were re-aligned and re-trimmed with only selected taxa, it is possible that very slight differences in alignment and trimming can cause differences in CRuMs monophyly in our dataset. We also noticed during our analyses that re-running the same dataset under the same evolutionary model could produce trees that differed in monophyly of CRuMs. This could be due to sensitivities to random starting seed number during ML estimation.

When CRuMs was not recovered, *Mantamonas* was always placed in the Obazoa phylum sister to apusomonad *Thecamonas.* Among single-gene trees where all relevant taxa were present, *Mantamonas* was sister to *Thecamonas* in 11/164 (trimAl) and 13/162 (BMGE) trees, roughly equal to the number of trees that recovered CRuMs (11/177 trimAl, 13/176 BMGE).

*Mantomonas* was retrieved from the EukProt v3 database (EP00003), as were other CRuMs members *Rigifila ramosa* (EP00004) and *Diphylleia rotans* (EP00002). *Thecamonas* was retrieved from Burki et al 2016. By BLASTP search of our *Thecamonas* proteins to other species in the EukProt database, 58/211 proteins had a top hit with other apusomonads, and 37/211 hit to *Choanocystis* sp. (Centroheliozoa, Haptista). *Mantamonas* proteins had 10/204 proteins with a top hit to other CRuMs, and other proteins had no tendency. We are not sure if this is a result of contamination in the *Thecamonas* dataset that might influence the placement of *Thecamonas*/*Mantamonas* in our trees – however, no single-gene trees place *Thecamonas* and *Choanocystis* sp. together.

Regardless, as the monophyly of Obazoa, Amoebozoa, CRuMs and Malawimonada is recovered in all of our trees with perfect support, we do not think that potential issues with these two taxa influence the main focus of the study, which was to examine the placements of Telonemia with Hemimastigophora, SAR, Haptista and Provora, particularly the influence of the deep-branch Telonemia sp. DSEL18 on these topologies.


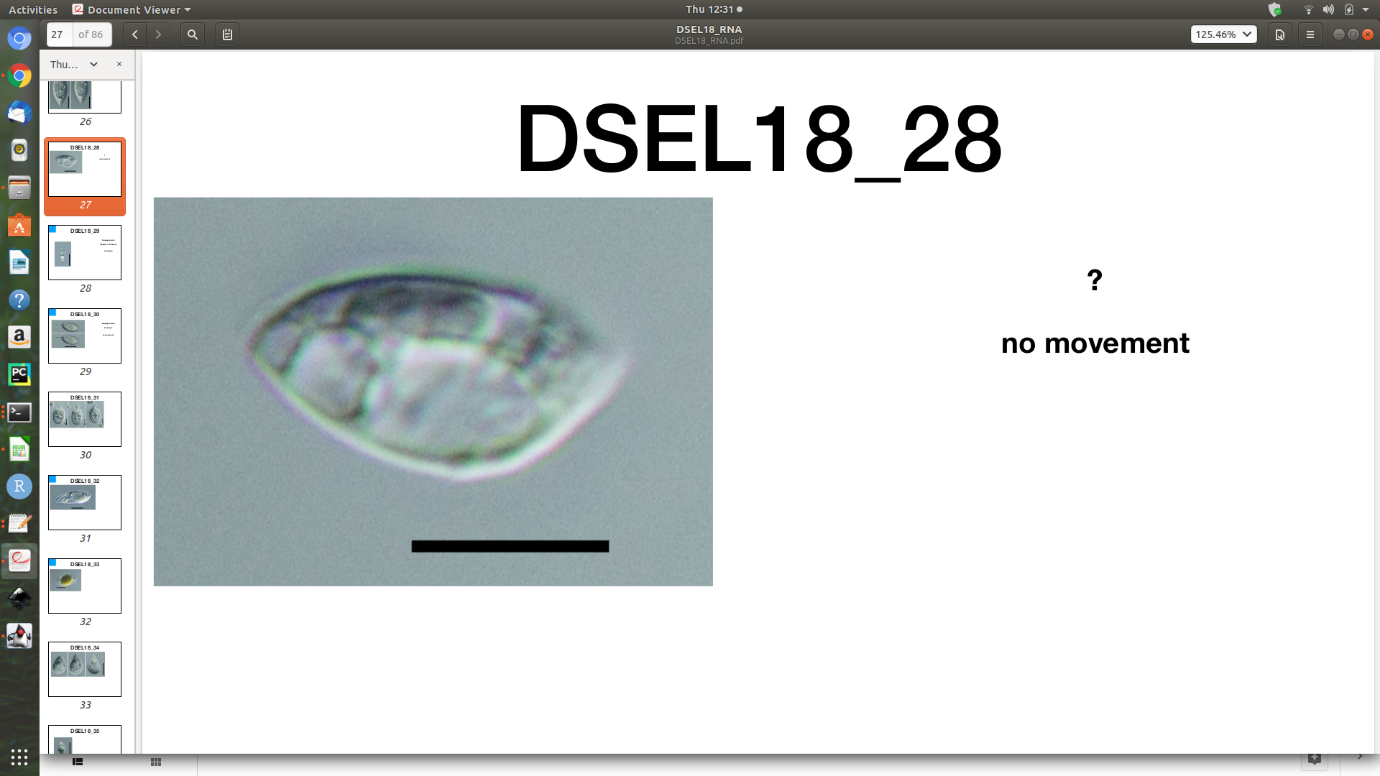


**Fig S1.** Light micrograph of Telonemia sp. DSEL18, from which transcriptome data was obtained. No cell movement was observed. Scale bar depicts 50 µm.


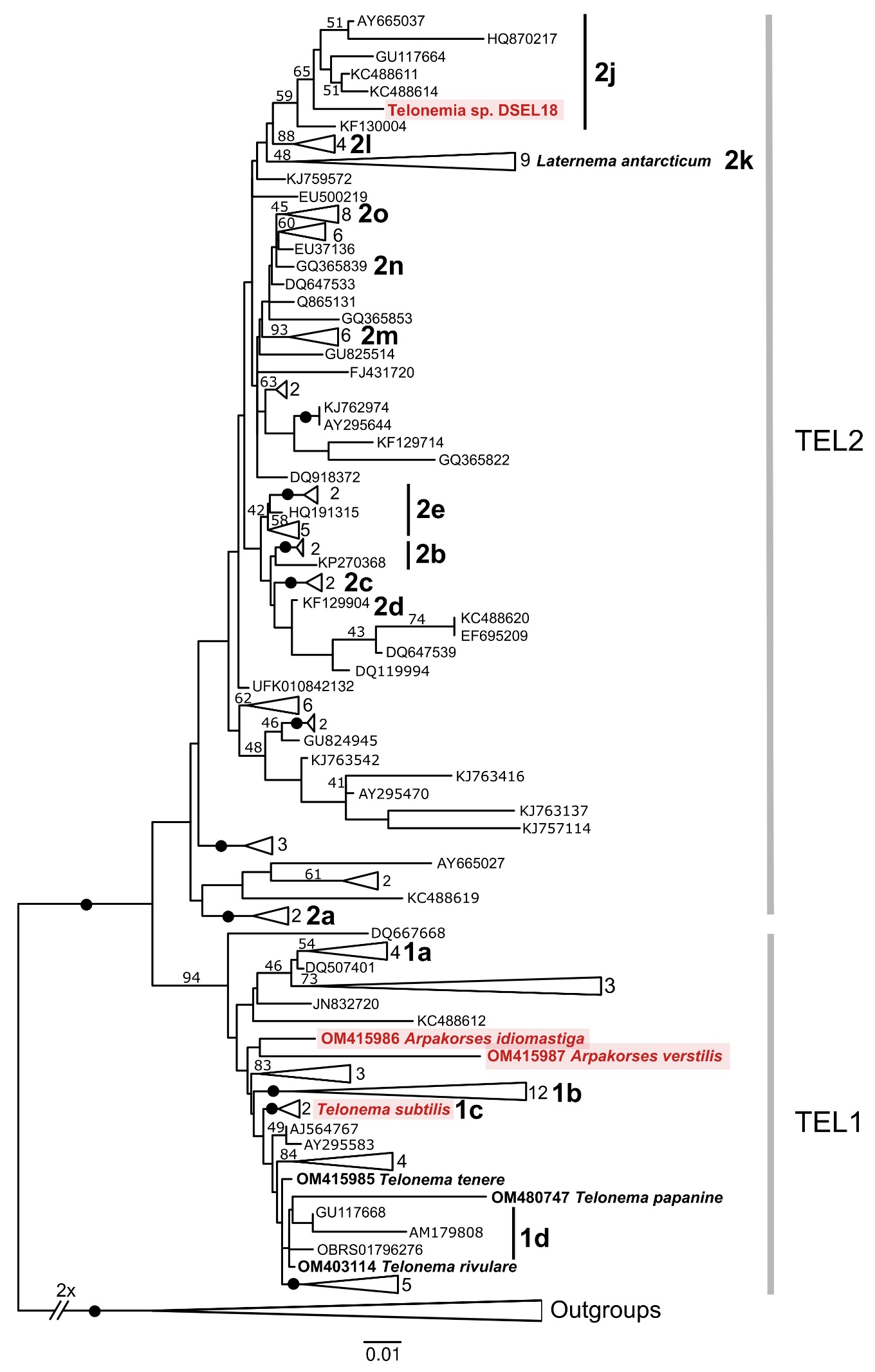


**Fig S2**. Maximum-likelihood phylogeny of small subunit ribosomal RNA (SSU rRNA) inferred under model TN+F+R3 with 1,000 non-parametric bootstraps. Telonemid species with multi-gene data available are coloured red. Circles on branches represent non-parametric bootstrap support ≥ 90%, while bootstrap support values < 50% are not shown. Clades with bootstrap support > 50% are collapsed, except for the clade containing Telonemia sp. DSEL18, and labels in bold are sub-groups originally described by (Bråte et al. 2010) and again recovered by (Tikhonenkov et al. 2022a). Number of sequences in collapsed clades are given as numbers to the right of clades. The branch containing outgroups has been shortened to half its original length. Scale bar represents estimated nucleotide substitutions per site.


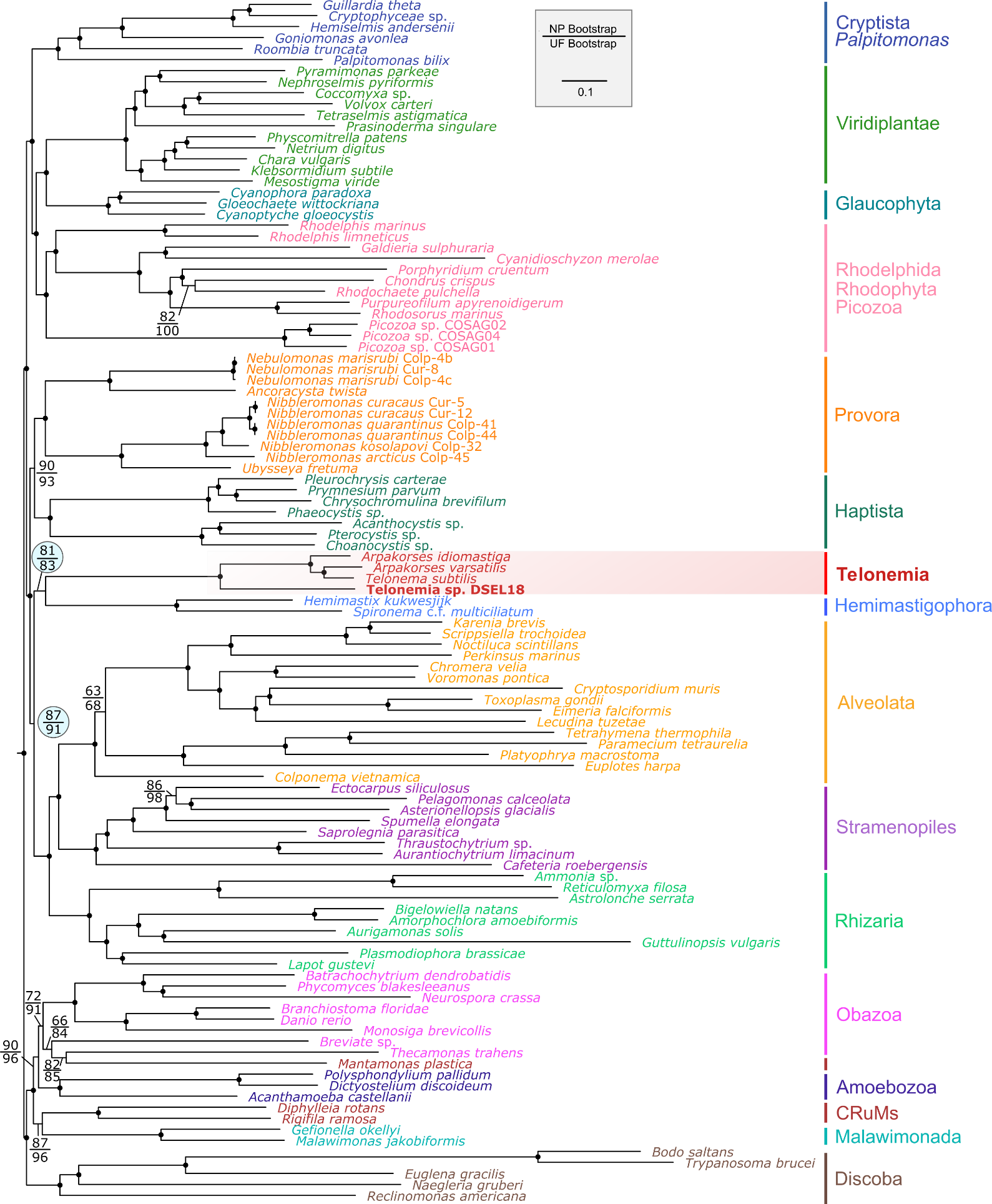


**Fig S3.** Maximum-likelihood tree inferred from 233 trimAl-trimmed proteins and 109 taxa (73,229 sites) under the LG+C60+F+R+PMSF model of evolution, with LG+C20+F+R used as the guide tree. Node values show non-parametric bootstrap support out of 100 (top) and ultra-fast bootstrap support out of 1,000 (bottom). Circles represent ultra-fast bootstrap support of ≥ 98% and non-parametric bootstrap support of ≥ 90%. Scale bar represents estimated amino acid substitutions per site.


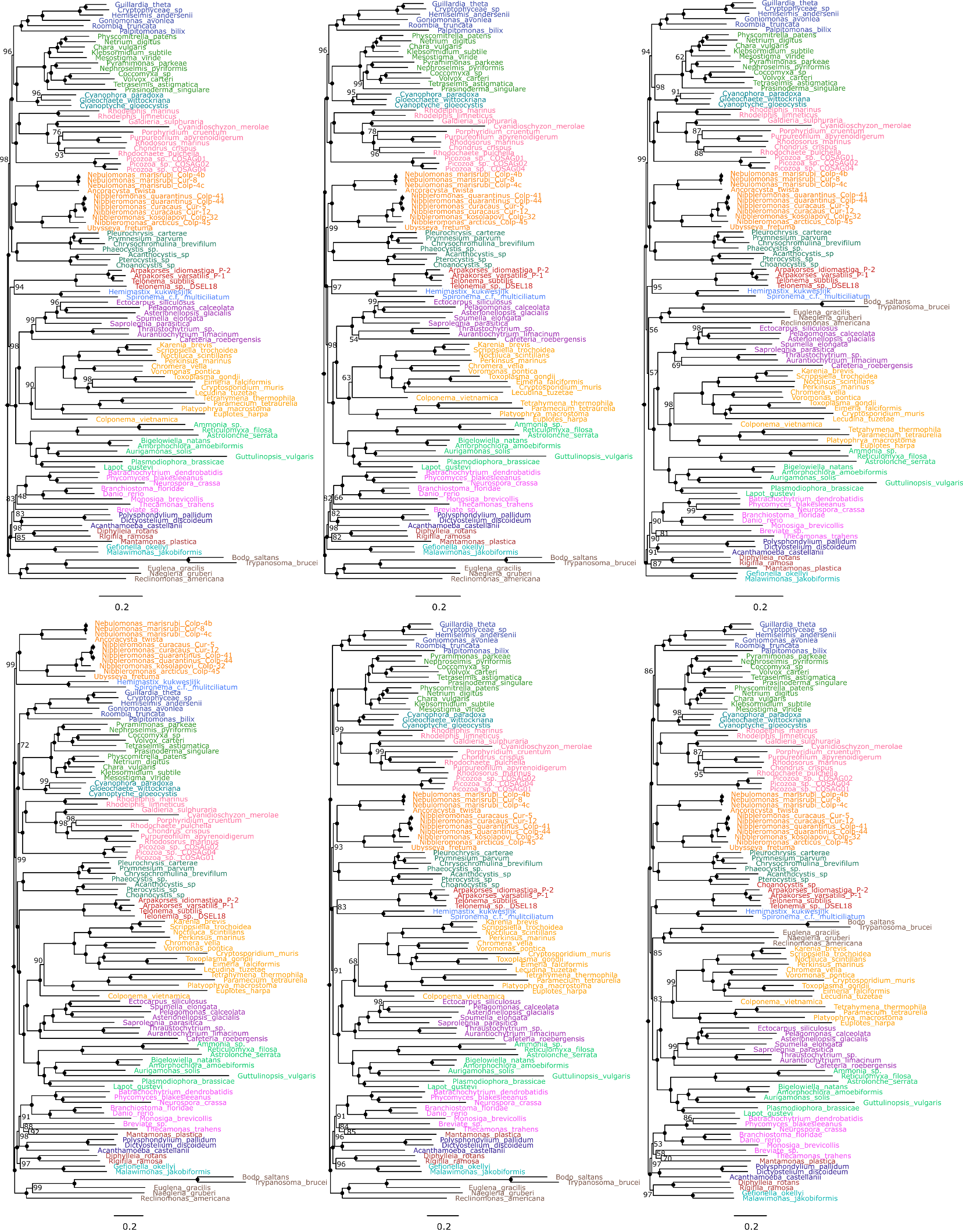


**Fig S4.** ML trees of 262 (left), 233 (centre) and 192 (right) BMGE-trimmed (top) and trimAl-trimmed (bottom) proteins, including all telonemids. Node values show ultra-fast bootstrap support out of 1,000. Circles on nodes represent UF bootstrap support of 100%. Scale bar indicates substitutions per site. Used for Figure 2A.


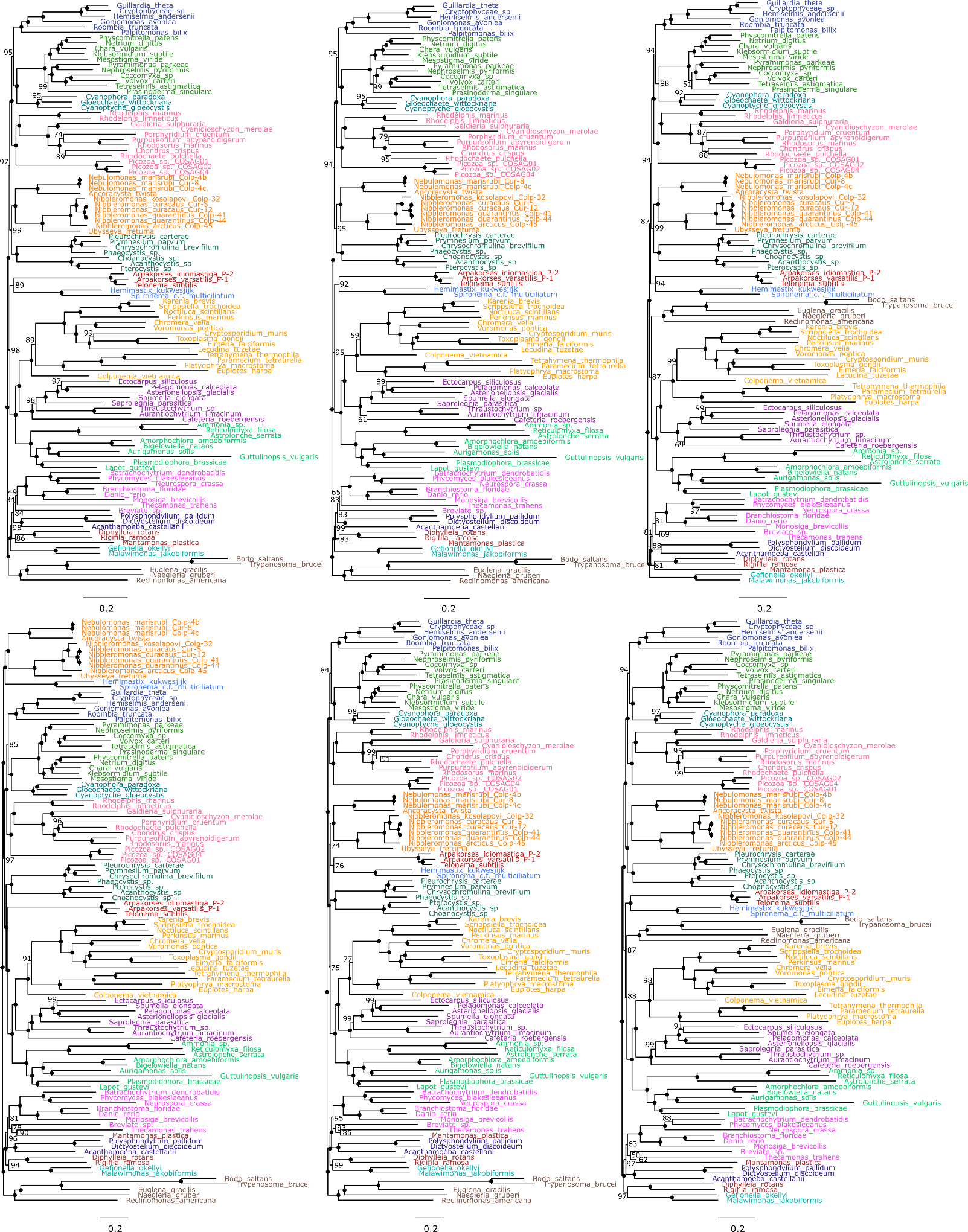


**Fig S5.** ML trees of 262 (left), 233 (centre) and 192 (right) BMGE-trimmed (top) and trimAl-trimmed (bottom) proteins, excluding Telonemia sp. DSEL18. Node values show ultra-fast bootstrap support out of 1,000. Circles on nodes represent UF bootstrap support of 100%. Scale bar indicates substitutions per site. Used for Figure 2A.

**
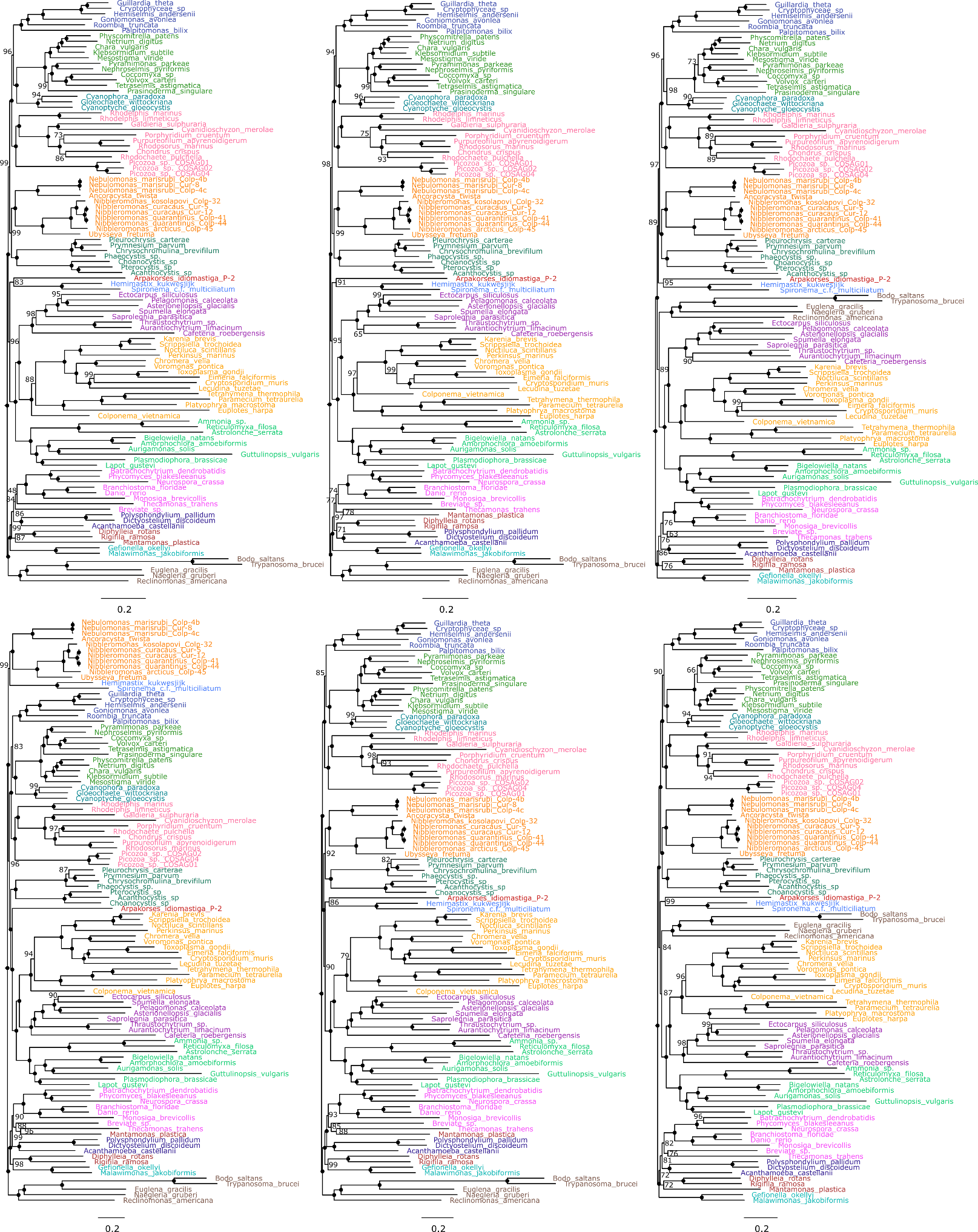
**

**Fig S6.** ML trees of 262 (left), 233 (centre) and 192 (right) BMGE-trimmed (top) and trimAl-trimmed (bottom) proteins, including only one telonemid with the highest coverage in alignments. Node values show ultra-fast bootstrap support out of 1,000. Circles on nodes represent UF bootstrap support of 100%. Scale bar indicates substitutions per site. Used for Figure 2A.


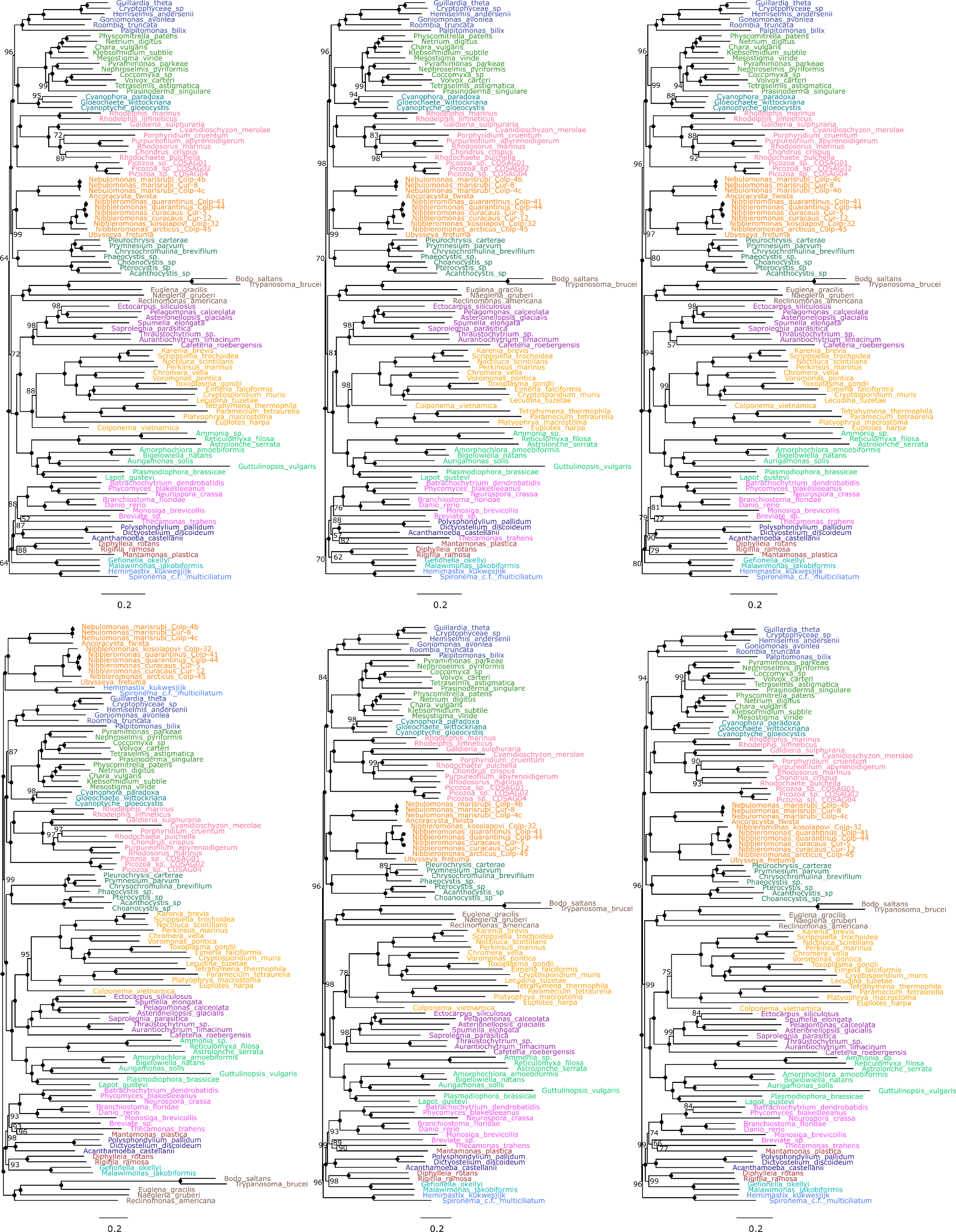


**Fig S7.** ML trees of 262 (left), 233 (centre) and 192 (right) BMGE-trimmed (top) and trimAl-trimmed (bottom) proteins, excluding all telonemids. Node values show ultra-fast bootstrap support out of 1,000. Circles on nodes represent UF bootstrap support of 100%. Scale bar indicates substitutions per site. Used for Figure 2A.


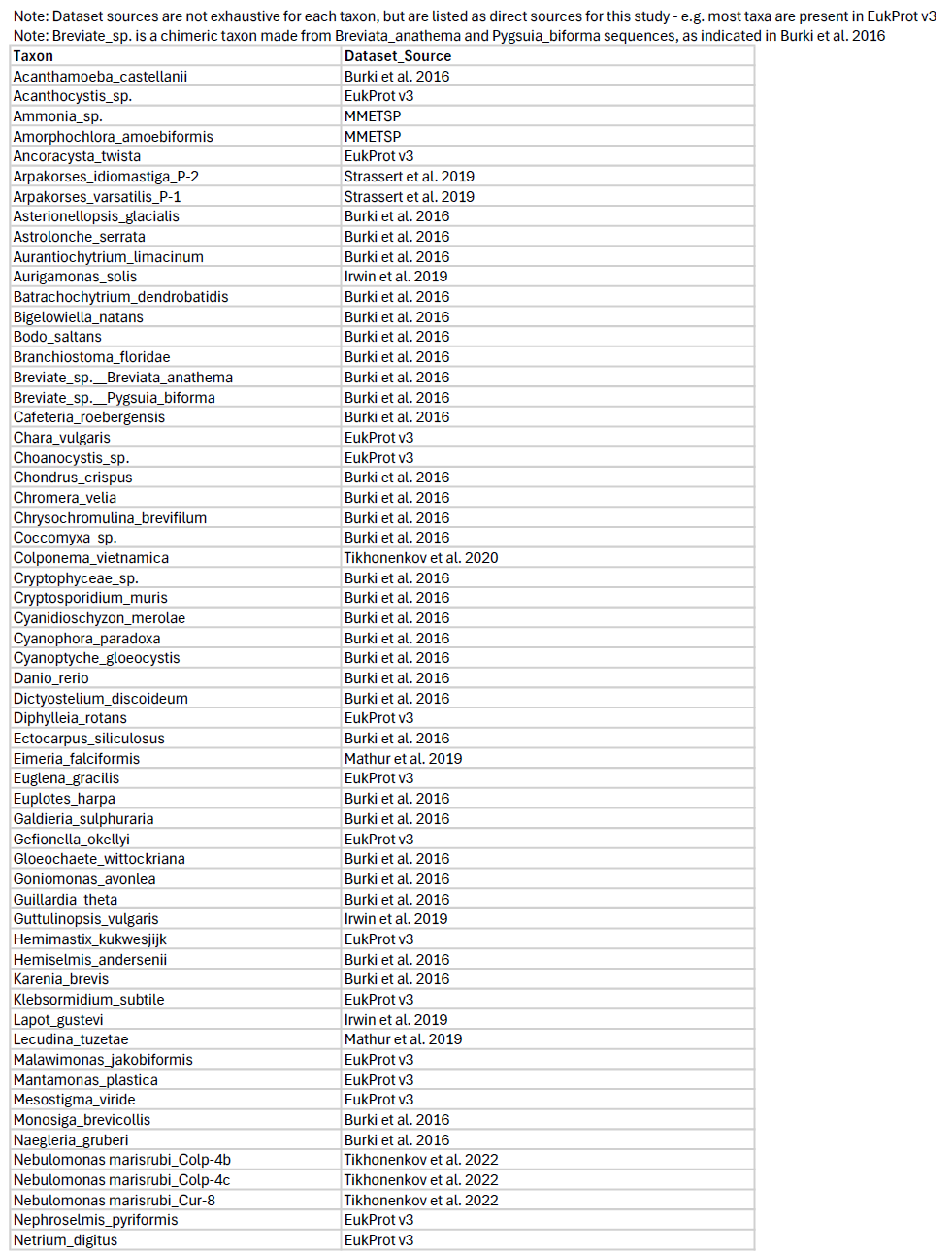


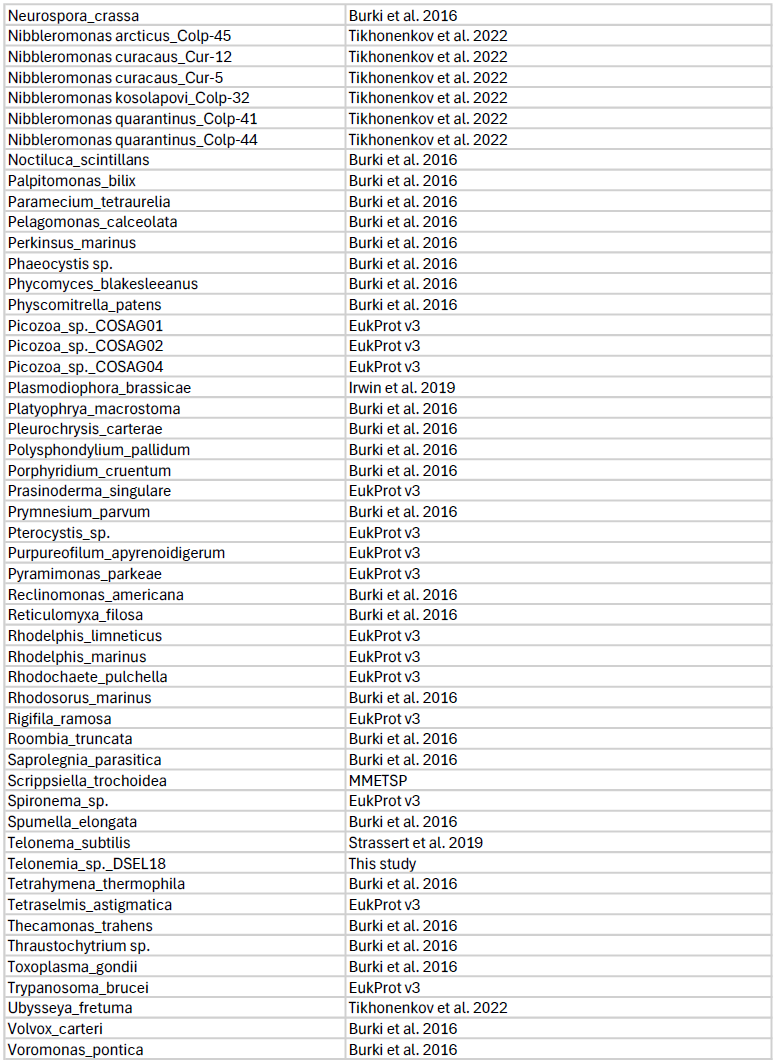


**Table S1.** Data sources used for this study.


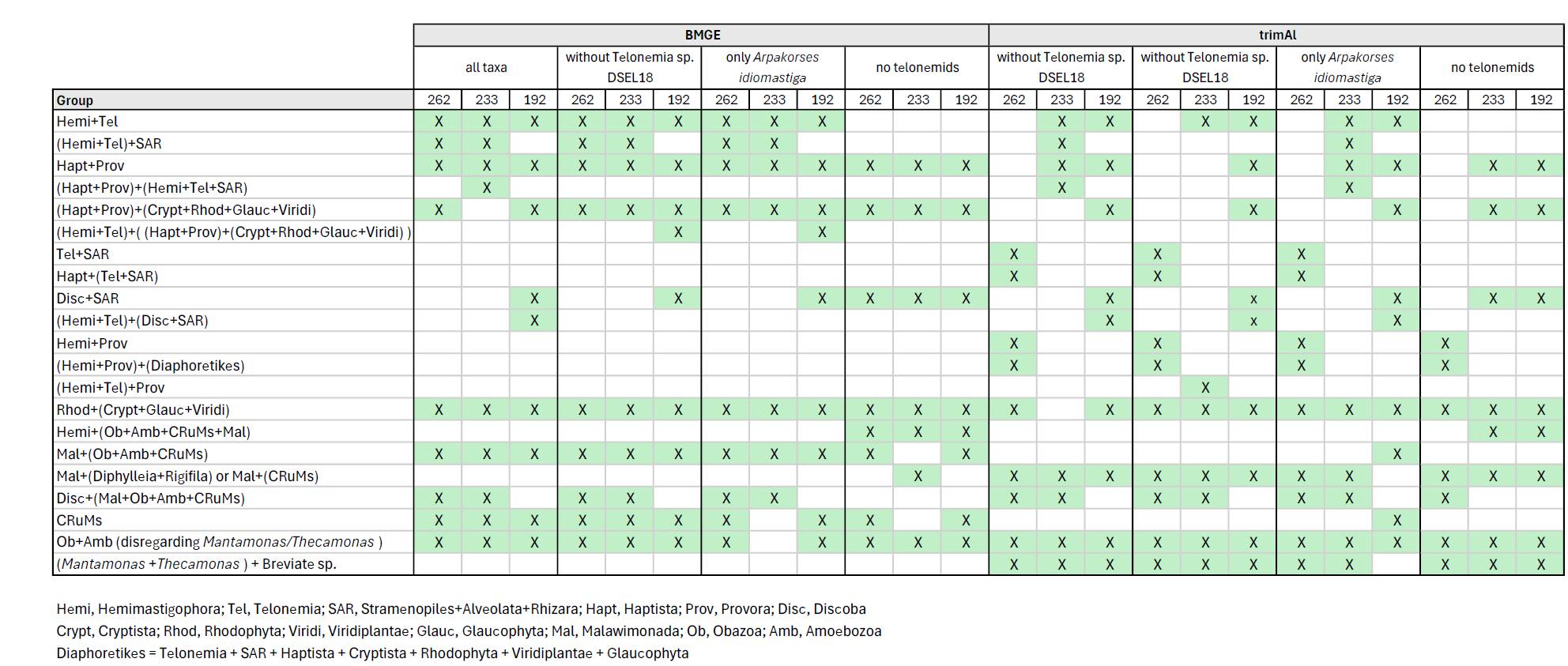


**Table S2.** Overview of various clades recovered from ML analyses of different gene sampling, telonemia sampling and trimming schemes (Fig 2A, Fig S4-S7).


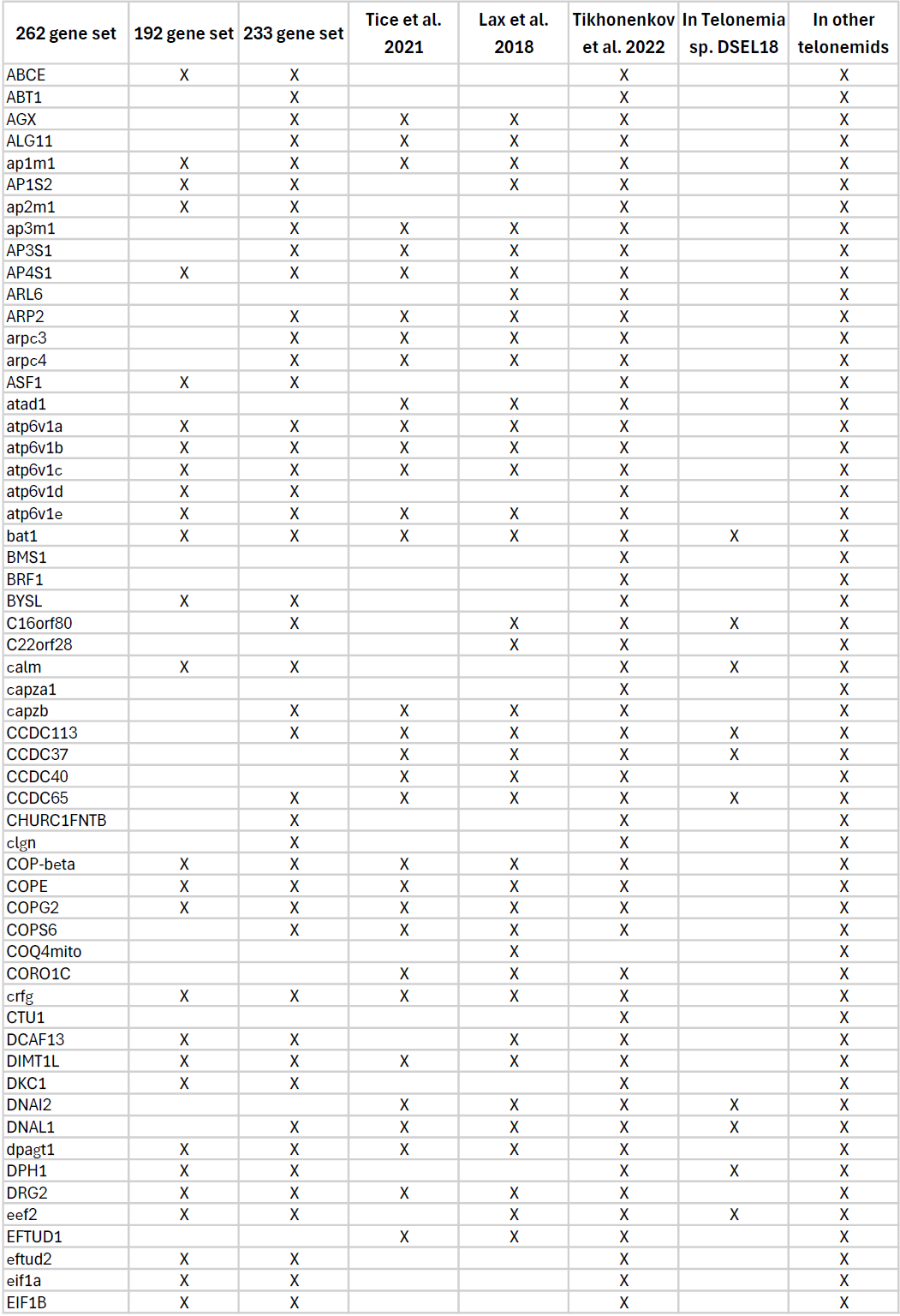


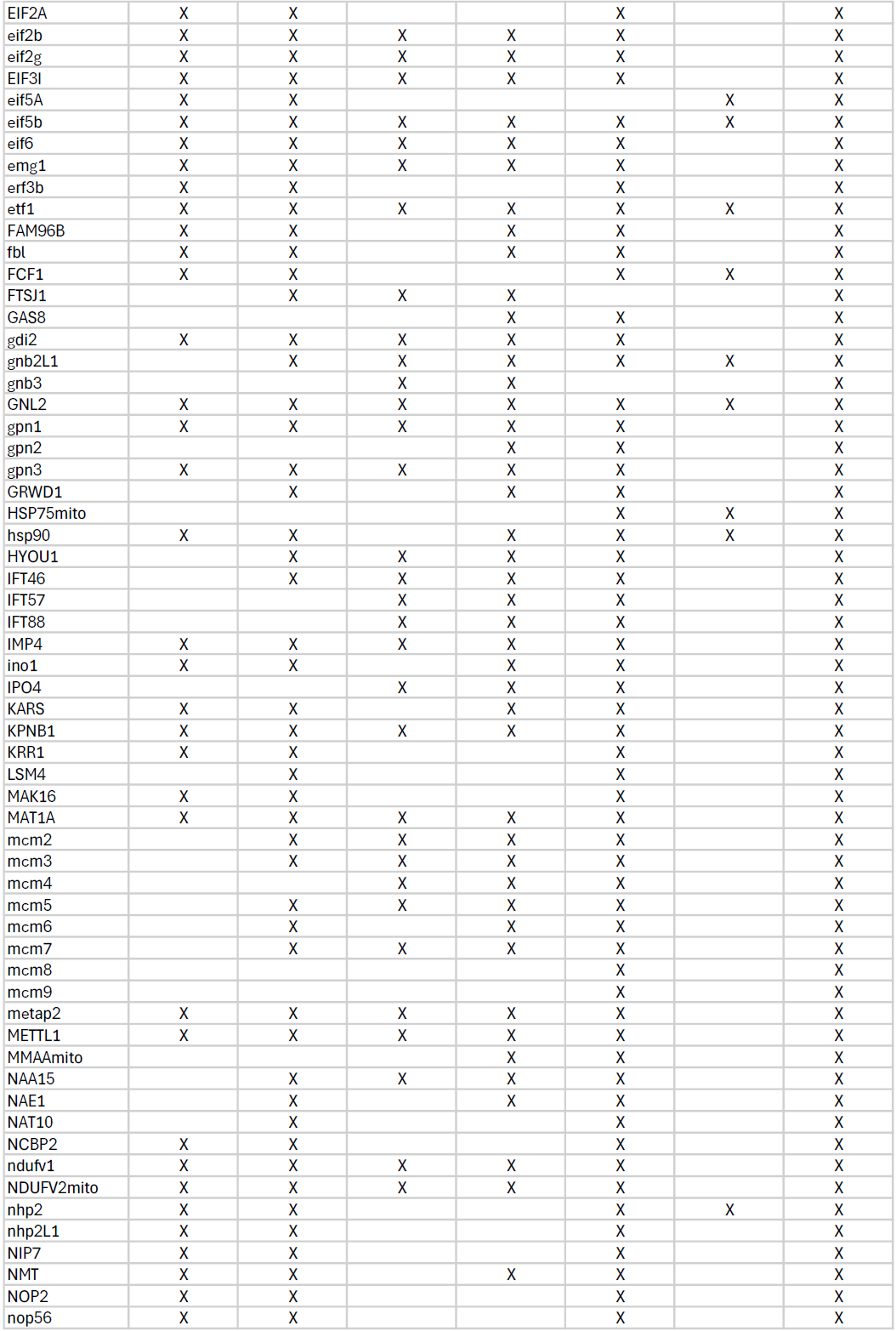


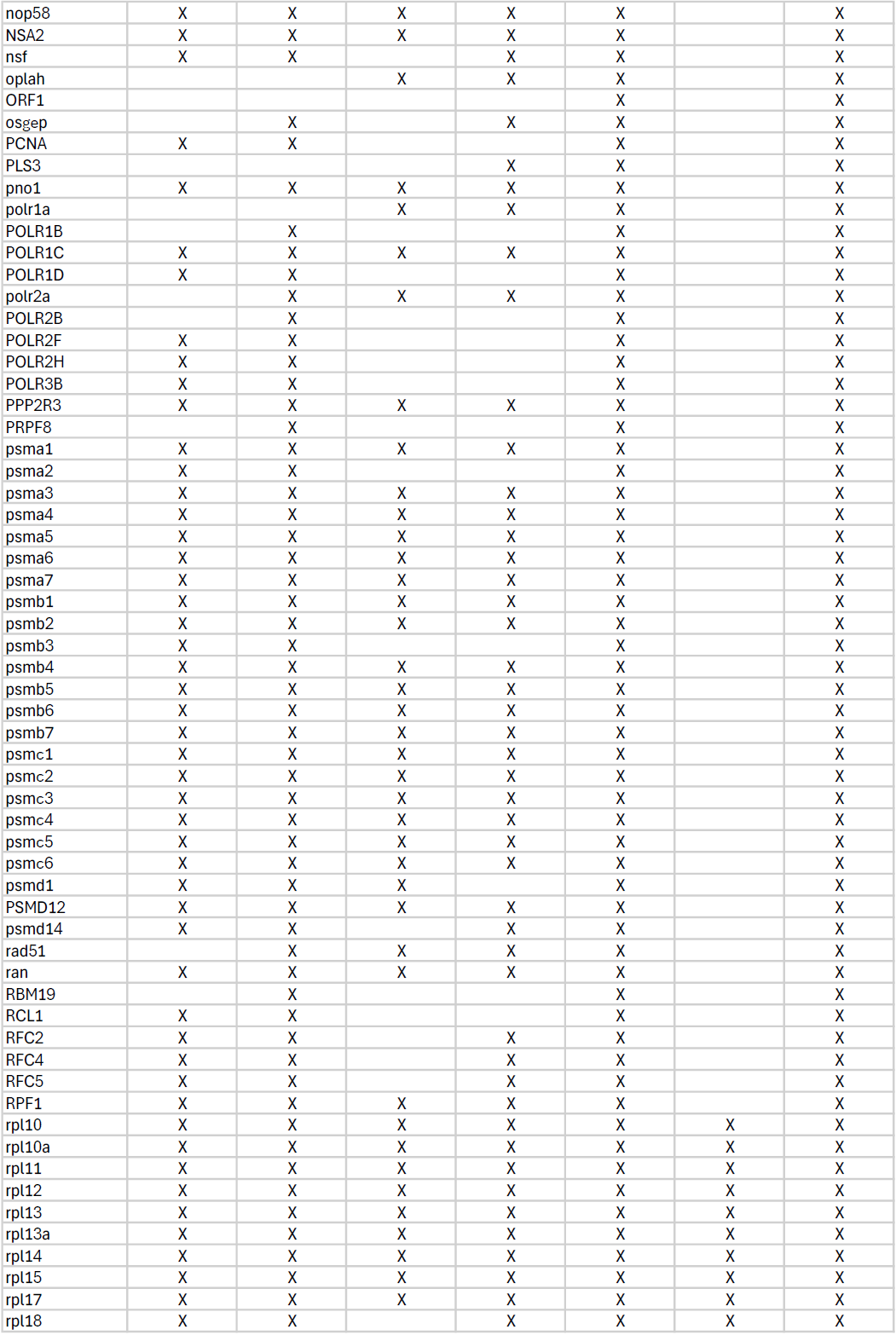


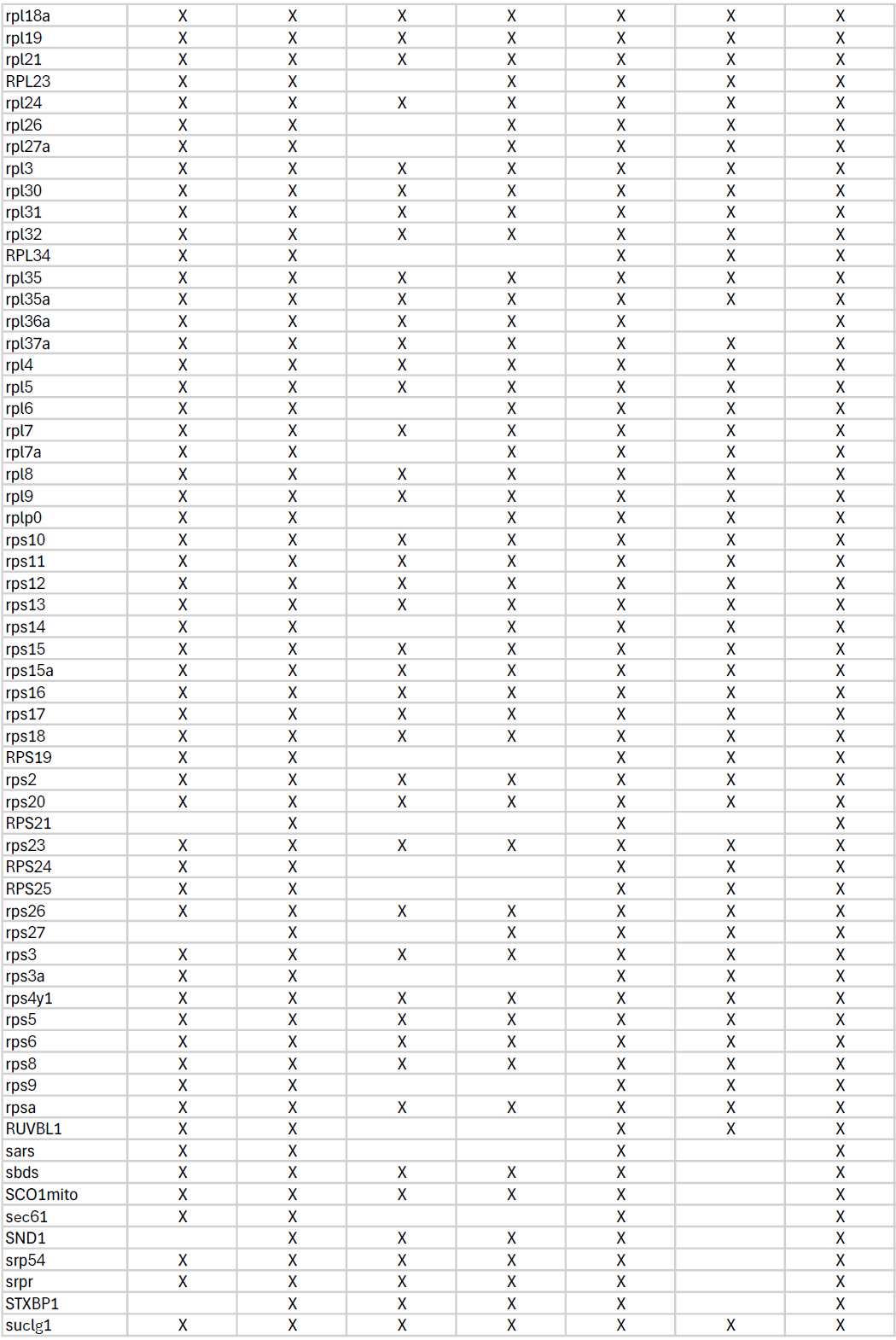


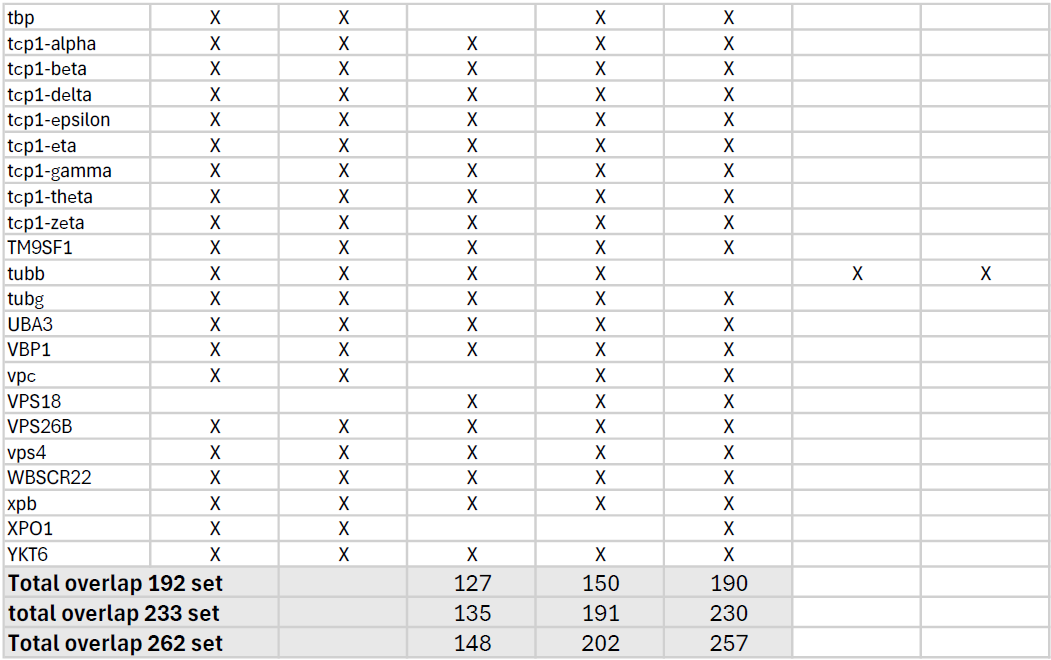


**Table S3.** Comparison of gene overlap from this dataset and those of Tice et al. 2021, Lax et al. 2018 and Tikhonenkov et al. 2022.


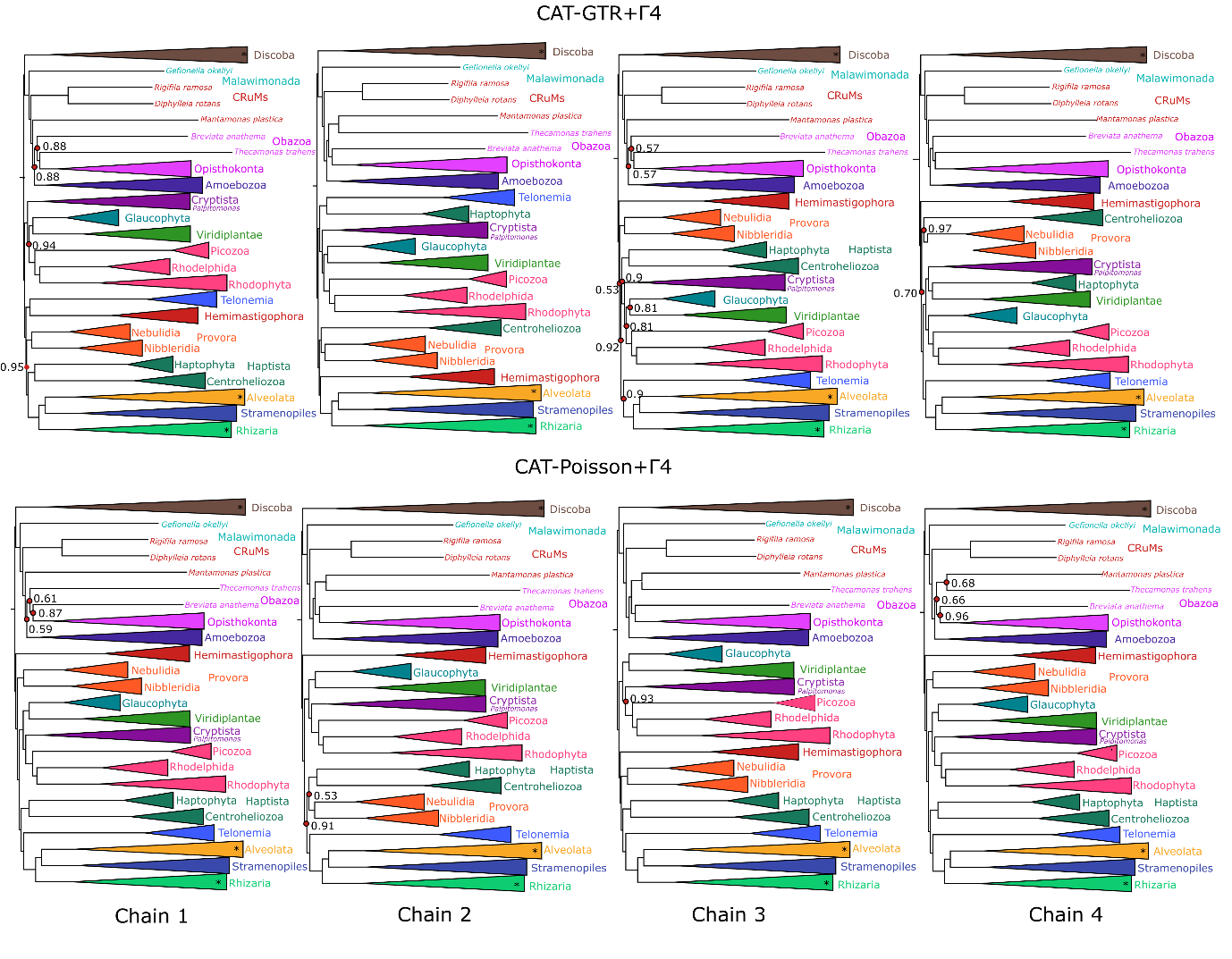


**Fig. S8.** Bayesian inference using a 233-protein, 81-taxa matrix inferred under the CAT-GTR+Γ4 (top row) and CAT-Poisson+Γ4 (bottom row) models of evolution. After four independent MCMC chains, each chain was summarized separately after 15,000 generations (CAT-GTR+Γ4) or 10,000 generations (CAT-poisson+Γ4) with 10% burn-in. Support values at red dots indicate posterior probability ≤ 0.98. Clades labelled with black stars have had branches scaled to half their original length.
